# BST2/Tetherin Inhibits Dengue Virus Release from Human Hepatoma Cells

**DOI:** 10.1371/journal.pone.0051033

**Published:** 2012-12-07

**Authors:** Xiao-Ben Pan, Jin-Chao Han, Xu Cong, Lai Wei

**Affiliations:** 1 Peking University People's Hospital, Peking University Hepatology Institute, Beijing Key Laboratory of Hepatitis C and Immunotherapy for Liver Diseases, Beijing, P.R. China; 2 Peking University People's Hospital, Department of Infectious Disease, Beijing, P.R. China; Nanyang Technological University, Singapore

## Abstract

Type I interferons (IFN) have been shown to play an important role for inhibiting Dengue virus (DENV) infection. Identifying IFN-induced cellular proteins are essential for understanding its mechanisms against DENV. Here we established stable Huh7-derived cell lines expressing the IFN-induced cell membrane protein BST2 (Huh7-BST2) or its variant bearing a V5 tag at the C-terminal (Huh7-BST5CV5). These cell lines were infected with DENV to determine proteins modulating their anti-DENV response. We found that expression of BST2 did not affect the efficiency of DENV infection and intracellular replication. Rather, it significantly reduced the virion yield of the infected cells, particularly at low MOI infection. In addition, BST2 also decreased the foci formation and the size of infectious foci in cultured Huh7 monolayers with media containing methocellulose. The addition of the V5 tag at C-terminal inhibited the GPI modification of BST2 and blocked its shift from endoplasm to cytoplastic membrane. BST2CV5 did not affect DENV infection and foci formation in Huh7 cells but reduced virion yield by 1 log at low MOI infection. Interestingly, intracellular BST2CV5 expression was reduced by high level of DENV production.

**Conclusion:**

Our results imply that BST2 is a functional mediator of the IFN response against DENV infection. BST2 inhibits the release of DENV virions from Huh7 cells and limits viral cell-to-cell transmission. BST2CV5 variant is unable to inhibit DENV release but impairs viral infection in cells.

## Introduction

Dengue virus (DENV) belongs to the family *Flaviviridae*, and DENV infection remains a global public health problem due to a lack of effective treatment or vaccine [1]–[3]. The World Health Organization estimates that at least 2.5 billion people are at risk of contracting dengue and the number of infections worldwide may reach 10 million cases per year [4]. Most infected patients experience dengue fever, but 2 to 20% of all cases manifest as dengue hemorrhagic fever, a severe and often lethal illness [5].

Although, DENV has been demonstrated to inhibit interferon (IFN) signaling in cells, this inhibition is attributed to several DENV proteins and pre-existing enhancing antibodies [6]–[8]. Type I IFN plays an important role in the pathogenesis of DENV infection. Mice with deficiencies in the type I IFNs production or JAK-STAT signaling pathway are susceptible to DENV infection [9]–[11]. Clinically, low levels of the IFN-α/β producing plasmacytoid dendritic cells have been observed in dengue hemorrhagic fever patients [12], [13]. Secretion of type I interferon by dendritic cells and mast cells contributes to the generation of antiviral innate and adaptive immune responses [14]–[16].

IFNs can alter the expression of hundreds of cellular genes [17]. Our group and others have previously demonstrated that the expression of IFN-inducible proteins, such as Viperin, IFITM2, IFITM3, double stranded RNA dependent protein kinase (PKR), and interferon-stimulated gene (ISG)-20, in HEK293 cells was able to inhibit DENV [18]–[21]. Most recently, ISG15 was demonstrated to play an anti-DENV function via protein ISGylation. ISG12b2 was identified as a novel inner mitochondrial membrane ISG that regulates mitochondria-mediated apoptosis during DENV infection [22], [23]. In our current study, we report that the expression of ISG BST2 (bone marrow stromal cell antigen 2), also known as CD317, HM1.24 or tetherin [24]–[26], plays a role in inhibiting DENVE production.

## Materials and Methods

### Plasmid construction and establishment of cell lines

Plasmid pcDNA5/FRT/BST2 was constructed as described previously [22]. To construct plasmid pcDNA5/FRT/BST2CV5, BST2 cDNA (Gene ID: 684) was amplified from pcDNA5/FRT/BST2 with a pair of primers (Forward: 5′-GAGCTTAAGATGGCATCTACTTCGTATGACTA-3′. Reverse: 5′-CACGCGGCCGCCCTGCAGCAGAGCGCTGAGGCCC-3′). The PCR product was digested with Afl II and Not I (New England Biolabs, Ipswich, MA, USA), and ligated with vector fragment recovered from Afl II and Not I digested pcDNA5/FRT/Viperin-CV5 [27]. To establish stable Huh7 cell lines that express BST2 (Huh7-BST2) and carboxyl-terminally V5 tagged BST2 (Huh7-BST5CV5), Huh7 cells (Institute of Cytology, Chinese Academy of Sciences, Shanghai, China) were transfected with plasmid pCDNA5/FRT/BST2CV5 or pCDNA5/FRT/BST2CV5 and pcDNA3 at a molar ratio of 9 to 1 and cultured with complete DMEM containing 250 µg/ml G418. G418-resistant cell clones that express BST2 or BST2CV5 were identified by detection of the desired proteins in cell lysates by western blot.

### Cell culture and virus infection

A cell-based flavivirus immunodetection (CFI) assay was used to determine the *in vitro* anti-dengue activity of BST2. Briefly, 2×10^4^ of parent Huh7, Huh7-BST2 or Huh7-BST2CV5 cells were seeded in 96-well plate for overnight before they were infected with DENV (serotype II, TSV01 strain) at the given multiplicity of infection (MOI) for 1 h [28]. Cells were incubated in complete Dulbecco's modified minimal essential medium (DMEM, Invitrogen, Carlsbad, CA) for 2 days.

### Indirect immunofluorescence and in-cell western immunoassay

Cells were fixed with PBS containing 2% paraformaldehyde and permeabilized with 0.1% triton X-100 PBS. Cells were blocked and then incubated with mouse monoclonal antibody against DENV E protein (1∶500 dilution, Clone D1-4G2-4-15, Billerica, EMD Millipore, MA) or rabbit polyclonal anti-BST2 antibody (1∶250 dilution, Proteintech, Chicago, IL). Bound primary antibody was visualized by Alexa Fluor 488-conjugated goat anti-mouse IgG or Alesa Fluor 594-conjugated goat anti-rabbit IgG (Invitrogen, Carlsbad, CA). Cell nuclei were stained with DAPI (4′,6-diamidino-2-phenylindole, Invitrogen).

In-cell western immunoassay was performed as previously described [27]. Primary antibodies were bound with an anti-mouse IRDye 800CW-labeled secondary antibody (green color) or anti-rabbit IRDye 700CW-labeled secondary antibody (red color). Cell viability was determined by Sapphire 700 staining (Red color). The fluorescence signal intensity was quantified with LI-COR Odyssey Infrared Imaging System (LI-COR Biotechnology, Lincoln, NE).

### Western blot

Expression levels of BST2 and its variant in the cell lines were evaluated using western blot by comparing parental Huh7 cells treated with 0 to 3000 IU/ml of IFN-α for 48 h. Whole cell monolayers were washed once with phosphate-buffered saline buffer and lysed with 1×sodium dodecyl sulfate (SDS) Sample Buffer. For cell fractional protein analysis, membrane and cytosol fractions were separated by centrifugation methods by using of a subcellular protein fractionation kit (Thermo Scientific, Rockford, IL). A fraction of the cell lysate was separated on sodium dodecyl sulfate 12% SDS polyacrylamide gels and electrophoretically transferred onto a polyvinylidene difluoride membrane (PVDF, EMD Millipore). The proteins on membrane were bound with indicated antibodies and detected by Odyssey Infrared Imaging System (LI-COR Biotechnology) as described above.

### Determination of infectivity titer

Infectivity titers were determined by using an earlier described protocol [29]. Naive Huh7 cells (2×10^4^) were plated per well in a 96-well plate the day before inoculation with 10-fold dilutions of cell culture supernatants in replicates of six for 2 days. Primary antibody for development was anti-DENV E protein (1∶500 dilution, Clone D1-4G2-4-15, EMD Millipore). Wells were scored positive if one or more cells were infected, and the TCID50 value was calculated. The experiment was performed in 3 replicates to generate statistically sufficient data.

### Infectious foci count

Cells were seeded into 24-well plate at a density of 2×10^5^/well (100% confluence) over night. Cells were infected with DENV at different MOI in replicates of six for 1 hour, and culture media were removed and replaced with media containing 0.5% methocellulose prevent cell-free virus infection. Two days after infection, cells were fixed and infected cell foci were revealed by In-Cell Western assay or indirect immunofluorescence. Quantitative analyses of 100 foci from each cell line were performed to reveal the average number of DENV-positive cells per focus. The experiment was performed in 3 replicates to generate statistically significant data.

### RNA quantification by qRT-PCR

Cellular RNA or viral RNA in culture medium were extracted using TRIzol Reagent (Invitrogen) or QIAamp viral RNA minikit (Qiagen, Valencia, CA) and reverse transcribed using SuperScript III (Invitrogen). Quantitative PCR (qPCR) reaction was performed on the ABI 7500 thermocycler (Applied Biosystems, Foster City, CA). The probe for DENV was 6-carboxyfluorescein [FAM]-5′-AGCATCATTCCAGGCAC-3′-MGBNFQ (molecular-groove binding nonfluorescence quencher; Applied Biosystems), forward primer 5′-GARAGACCA GAGATCCTGCTGTCT-3′ and reverse primer 5′-ACCATTCCATTTTCTGGCGTT-3′ (SBS Genetech, Beijing, China). The standard curve was generated using serial 10-fold dilutions of in vitro transcribed full-length DENV RNA (TSV01 strain). The housekeeping gene β-actin was used as control for normalization of DENV quantification. The probe for β-actin was FAM-TTCACCACCACGGCCGAGC-TAMRA, forward and reverse primers were ACCGAGCGCGGCTACAG and CTTAATGTCACGCACGATTTCC, respectively(SBS Genetech, Beijing, China).

## Results

### Expression and localization of BST2 and BST2CV5 in Huh7 cells

**Figure 1:**
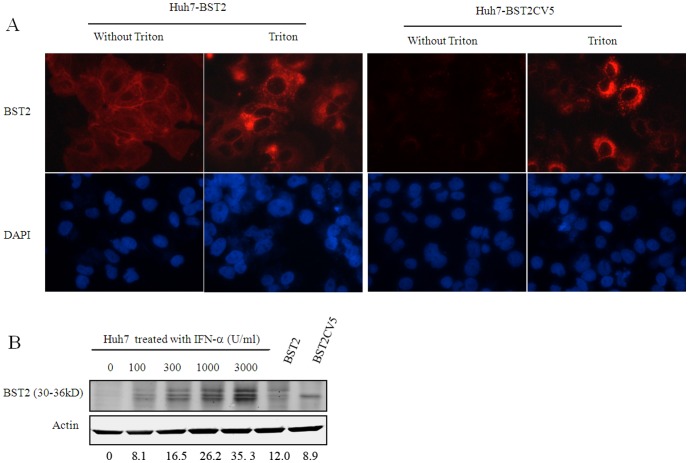
Expression and subcellular localization of BST2 and BST2CV5 proteins in Huh7 cells. (A). Huh7-BST2 and Huh7-BST2CV5 cells were fixed PBS containing 2% paraformaldehyde and left untreated or permeablized by incubation with PBS containing 0.1% Triton X-100. Cells were then blocked and incubated with a rabbit polyclonal anti-BST2 antibody (Proteintech). Bound primary antibody was visualized by Alesa Fluor 594-conjugated goat anti-rabbit IgG (Invitrogen, Carlsbad, CA). Cell nuclei were stained with DAPI. (B) Expression of BST2 expression in the lysates of parental Huh7 cells that were either left untreated or treated with the indicated concentrations of IFN-α for 24 h and cell lines Huh7-BST2 and Huh7-BST2CV5 was detected by a Western blot assay with a rabbit anti-BST2 antibody. β-actin served as a loading control. C. Cell lines were fractionated into membrane and cytoplasmic fractions, and each fraction was analyzed by Western blotting. The indicated gray values of the BST2/BST2V5 bands were quantified by using of an Odyssey Infrared Imaging System (LI-COR Biotechnology) and adjusted according to loading control β-actin. The values represent average from 3 independent experiments.

Huh7 is a hepatocarcinoma cell line, which is highly permissive for DENV infection [30]. We established Huh7-derived stable cell lines that express wild-type or carboxy-terminally V5 epitope-tagged mutant BST2 proteins, designated as Huh7-BST2 and Huh7-BST2CV5, respectively. As shown in Fig. 1, expression of BST2 and its variant in the two cell lines were confirmed by indirect immunofluorescent staining and western blot. Consistent with a previous report [26], wild-type BST2 was observed in cells with or without detergent-permeablizing treatment. This suggests that BST2 localizes both in the cytoplasm and plasma membrane. In marked contrast, the C-terminal V5-tagged BST2 protein (BST2CV5) was only observed in the presence of detergent-permeablizing treatment and mostly detected in cytoplasm (Fig. 1.A and C). This suggests that the addition of 14 amino acid residues of V5 eiptope tag at the C-terminus of BST2 alters the intracellular trafficking of BST2 that prevents its expression on the cell surface. To evaluate the expression levels of BST2 and its variant in the cell lines, we compared their expression levels with that of parent Huh7 induced by various concentrations of IFN-α. The results showed that the expression of BST2 and BST2CV5 in cell lines were comparable to that of 100–300 U/ml IFN-α induction (Fig. 1.B).

### BST2 inhibits dengue infection at a post entry step

**Figure 2:**
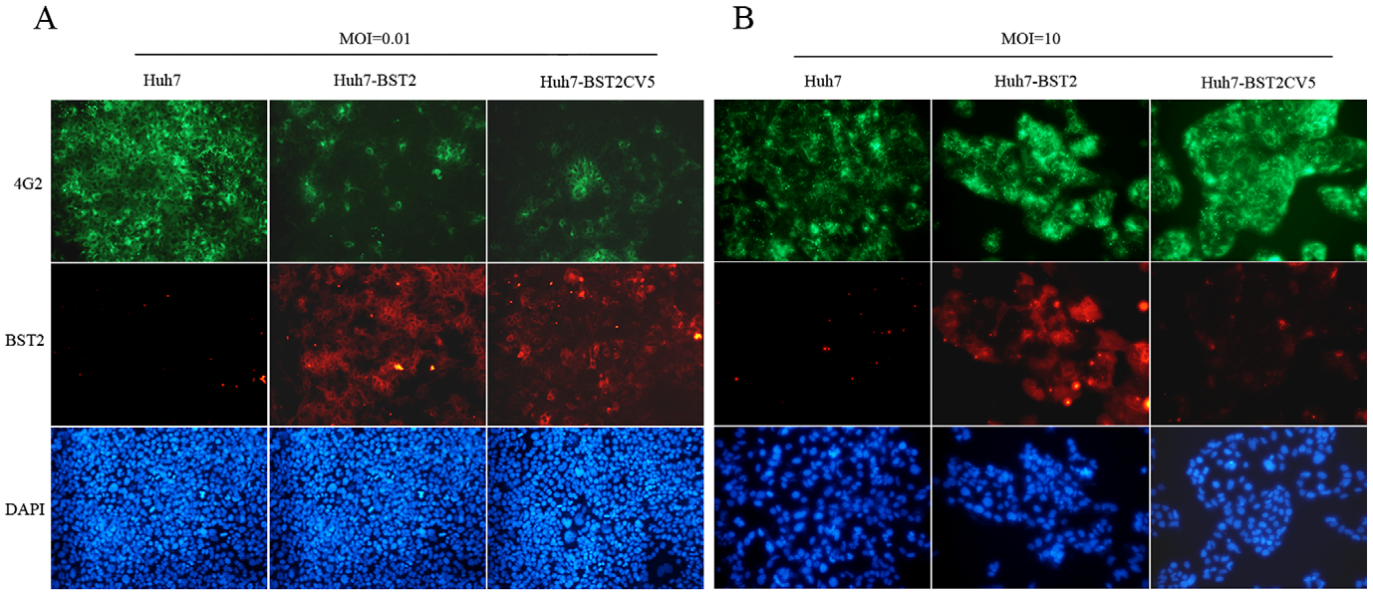
Immunofluorescent staining for DENV infection in Huh7-BST2 and Huh7-BST2CV5 cells. Cells were infected with DENV at indicated MOI and harvested on day 2. Cells were double-stained for DENV envelope protein 4G2 (top panel, green) and BST2 (middle panel, red). Cell nuclei were stained with DAPI (bottom panel, blue).

**Figure 3:**
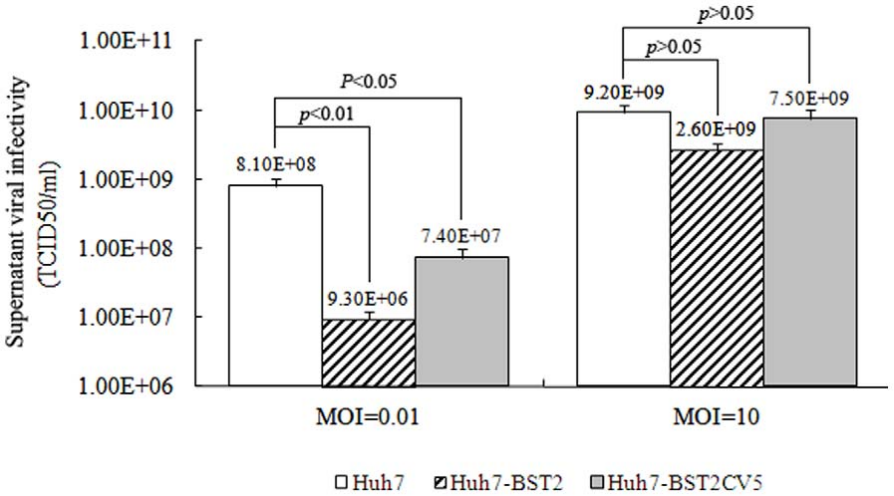
Viral infectivity detection of supernatant DENV in Huh7-BST2 and Huh7-BST2CV5 cells. The viral infectivity of supernatant DENV was determined by TCID50 method. The cells were infected with DENV at indicated MOI for 1 h; the media were replaced with complete media and cultured for 2 days. Dengue E protein was assayed by A cell-based flavivirus immunodetection assay. The values represent average from 3 independent experiments (n = 3). *p* values were calculated using Student's t test.

To test the effects of BST2 expression on DENV infection, parental Huh7, Huh7-BST2 and Huh7-BST2CV5 cells were infected with DENV at an MOI of 0.01 (Low) and 10 (High). While DENV infection was observed in the majority of parental Huh7 cells at low MOI infection on day 2, only a small fraction of Huh7-BST2 cells were infected (Fig. 2A). Using supernatant, DENV infectivity decreased by about 2 logs suggesting that at least one step of DENV replication cycle is inhibited in Huh7-BST2 cells (Fig. 3). Compared to wild-type BST2, expression of BST2CV5 demonstrated a weaker but still significant antiviral effect against DENV.

A high MOI (10) infection assay was performed to determine whether BST2 inhibits DENV entry in the cells. According to Poisson distribution, cells infected with DENV than one virus is calculated by formula *P*(>1) = 1−e^−10^(10+1) = 0.995. Therefore, such a multiplicity of infection will ensure that nearly 100% of the cells are initially infected with at least one infectious DENV virion. As shown in Fig. 2B, expression of BST2 and BST2CV5 did not inhibit DENV infection at high MOI. Interestingly, high MOI infection of DENV decreased the expression of BST2CV5 but not BST2. BST2 expression was able to decrease supernatant viral infectivity by about 25%, whereas no changes in intracellular viral infectivity were observed in the Huh7-BST2CV5 and parental Huh7 cells (Fig. 3).

### BST2 does not inhibit DENV replication

**Figure 4:**
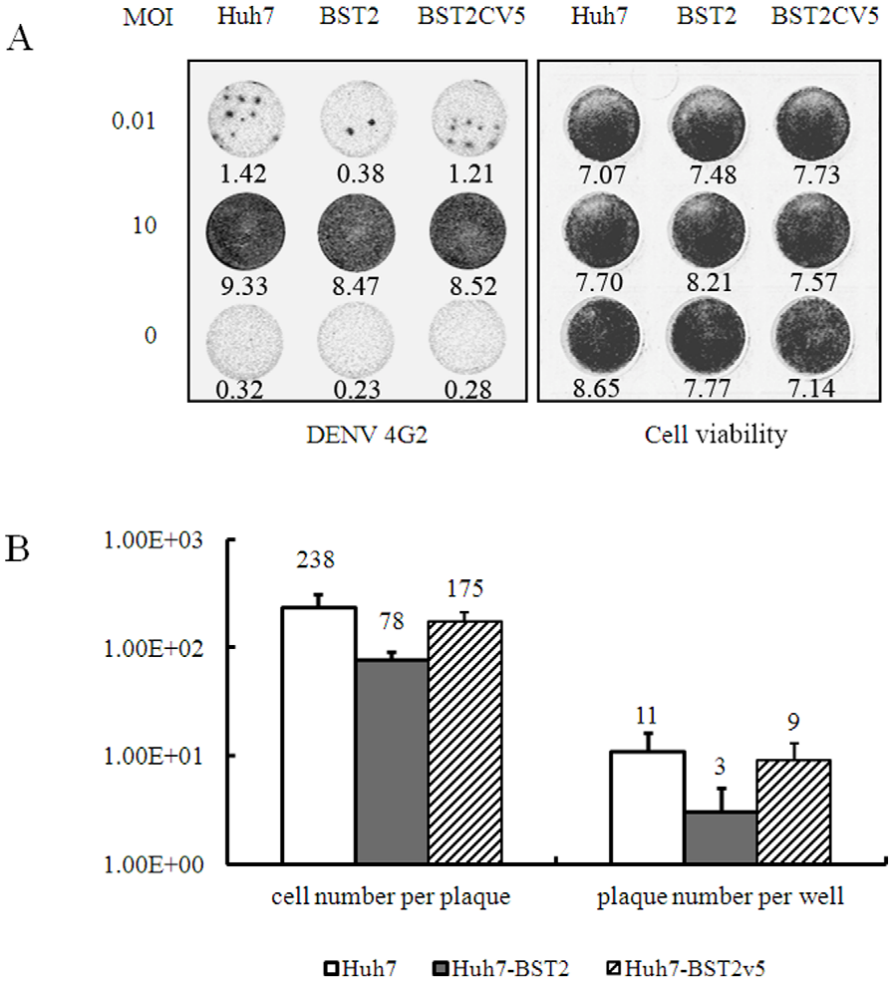
BST2 inhibits DENV spread via cell-to-cell transmission. The cells were infected with DENV at a MOI of 0.01 or 10 for 1 h and culture media were replaced with media containing 0.5% methocellulose to prevent cell-free virus infection and cultured for 2 days. (A) Representative DENV-infected cell foci from cultures of the three cell lines. The infected cell foci and cell viability were revealed by In-Cell Western assay by using of antibody against DENV E protein and Sapphire 700 staining, respectively. The indicated gray values of the dots were quantified by using of an Odyssey Infrared Imaging System (LI-COR Biotechnology). (B) The average infectious foci number per well in 24-well plate and the average DENV-infected cell number per focus from 100 foci were plotted. (C) The intracellular DENV RNA was determined for the cells infected with DENV at MOI of 10 by qRT-PCR assay. The values were presented as percentage of values from the Huh7-BST2 and Huh7-BST2CV5 cells compared with that from parent Huh7 cells. The experiment was performed in 3 replicates to generate statistically sufficient data. *p* values were calculated using Student's t test.

Infectious foci count and In-cell western blots were used to obtain an overall assessment of DENV spread and infection in these cell lines. In the foci count, free virus transmission is limited by 0.5% methocellulose in the medium. Among these three cell lines at high MOI infection, no obvious difference was observed in the intracellular DENV 4G2 protein and viral RNA levels (Fig. 4). This observation implied that BST2 and its variant did not inhibit DENV viral entry, viral replication, and protein translation.

**Figure 5:**
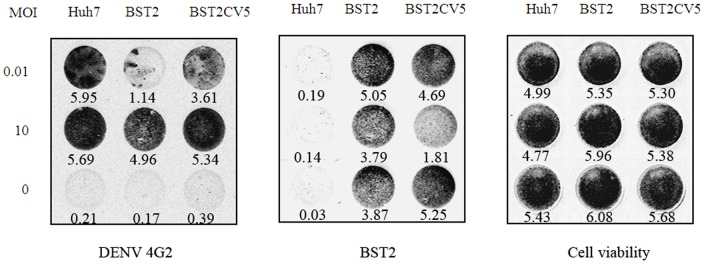
In-cell western analysis for DENV infection in Huh7-BST2 and Huh7-BST2CV5 cells. Cells were infected with DENV at indicated MOI and cultured for 2 days with complete medium. Cells were fixed and double-staining of DENV 4G2 protein and BST2 were revealed by In-Cell western assay. The indicated gray values of the dots were quantified by using of an Odyssey Infrared Imaging System (LI-COR Biotechnology). The values represent average from 3 independent experiments.

While DENV was freely transmitted in the infection system without restriction of 0.5% methocellulose, intracellular 4G2 protein markedly increased in all three cell lines at high MOI infection, whereas BST2 still moderately inhibited viral replication in Huh7-BST2 cells (Fig. 5). These results suggest that multiple rounds of infection from progeny virions occurred in this free transmission system that was partially inhibited by BST2.

### BST2 inhibits virion release and cell-to-cell transmission

The low MOI infection plot, as shown in Fig. 4A, shows the representative DENV-infected cell foci from the cultures of the three cell lines. The quantitative analysis showed that the infectious foci per well were decreased to about 30% by BST2 (Fig. 4B). However, BST2CV5 did not exert any effect. The average DENV-positive cell number per foci is 238 in Huh7 cells, whereas the cell numbers in the Huh7-BST2 and Huh7-BST2CV5 cell foci were 78 and 175, respectively. Altogether, the expression of BST2 but not BSTCV5 inhibited DENV release and cell-to-cell transmission in Huh7 cells.

## Discussion

BST2 is a transmembrane protein that contains a short N-terminal cytoplasmic domain, a membrane-spanning alpha-helix, a coiled-coil ectodomain, and a C-terminal GPI anchor [31]. This antiviral protein localizes at the plasma membrane as well as the membranes of multiple intracellular vesicles, including endosomes and the trans-Golgi network [32], [33]. At the plasma membrane, BST2 is found within cholesterol-enriched lipid rafts, presumably due to its C-terminal GPI modification. This optimally positions BST2 to interfere directly with virion release, since several lipid-enveloped viruses, including HIV-1 and Ebola, bud selectively from raft domains [34]–[38]. Consistent with these reports, our results showed that BST2 localizes to both the cell membrane and cytoplasm. The addition of the V5 tag at the C-terminus of BST2 demonstrated an altered intracellular distribution (Fig. 1). Furthermore, similar as previous report [25], [39], we found that three bands of BST2 distributed in the range from 30 to 36kd by western blot. We supposed that the different level of modification of BST2 likely cause the different size of BST2. However, for BST2V5, a single band of BST2 was observed and subcellular distribution of BST2 was changed. These results suggests that the addition of 14 amino acid residues of V5 eiptope at the C-terminus prevents modification of the GPI anchor.

BST2 potently inhibits the release of many enveloped viruses, including all retroviruses as well as members from five other families, including Filoviridae (Ebola and Marburg viruses), Arenaviridae (Lassa fever virus), Herpesviridase (Kaposi's sarcoma–associated herpesvirus) and Rabdoviridae (Vesicular stomatitis virus) and Flaviviridae (Hepatitis C virus) [26], [40]–[44]. It has been shown that BST2 tethers budding virions on the cell surface, which are subsequently endocytosed and degraded in the lysosomes [26]. BST2 can inhibit cell-to-cell transmission of HIV [45], [46]. However, interestingly, recent report also showed that BST2 enhanced HCMV entry into monocytic THP-1 cells. This might promote cell-to-cell transfer of HIV under some circumstances [47], [48]. In this study, we demonstrate that BST2 expression did not effect viral replication and entry in Huh7 cells at high MOI infection (Fig. 2B and Fig. 4). However, supernatant viral infectivity detection showed that BST2 inhibited DENV production (Fig. 3). Infectious foci assays strongly implied that BST2 expression markedly inhibits mature virions budding and cell-to-cell transmission (Fig. 4).

The addition of the V5 tag at the C-terminus of BST2 altered its intracellular distribution (Fig. 1). This suggests that the addition of the V5 tag likely impede C-terminal GPI anchor modification that is responsible for its enrichment in lipid rafts [25]. Although it demonstrated a weak antiviral activity in comparison with wild-type BST2, BST2CV5 was still able to inhibit DENV production (Fig. 2A, Fig. 3 and Fig. 5). Similar to BST2, BST2CV5 expression did not effect DENV RNA replication and viral entry. However, unlike BST2, BST2CV5 did not inhibit foci formation at low MOI infection (Fig. 4). This observation suggested that BST2CV5 was not able to inhibit cell-to-cell transmission of DENV in Huh7 cells. Furthermore, both immunofluorescence staining and In-cell western blot analysis showed high levels of DENV replication in Huh7 cells decrease the levels of intracellular BST2CV5, but not BST2 (Fig. 2B and Fig. 5). High DENV production decreased intracellular BST2CV5 levels supports our proposed hypothesis that BST2CV5 likely incorporates into DENV virion and excretes via virus budding [32]. Owing to the direct interaction between BST2 and the virion [49]–[51], this observation also suggests that sufficient amount of BST2 is indispensable to efficiently tether virus, especially a high yield virus like DENV. Indeed, this observation also addresses the issue whether a physiological induction of BST2 is sufficient and effective to inhibit DENV release *in vivo*.

In summary, although the additional studies are needed to explore the detailed mechanism, our results demonstrate that BST2 is an integral part of the host innate defense components against DENV infection in Huh7 cells.
